# DNA damage induces nucleoid compaction via the Mre11-Rad50 complex in the archaeon *Haloferax volcanii*

**DOI:** 10.1111/mmi.12091

**Published:** 2012-11-30

**Authors:** Stéphane Delmas, Iain G Duggin, Thorsten Allers

**Affiliations:** 1 School of Biology, University of Nottingham, Queen's Medical CentreNottingham, NG7 2UH, UK; 2 ithree Institute, University of TechnologyPO Box 123, Broadway, Sydney, NSW, 2007, Australia

## Abstract

In prokaryotes the genome is organized in a dynamic structure called the nucleoid, which is embedded in the cytoplasm. We show here that in the archaeon *Haloferax volcanii*, compaction and reorganization of the nucleoid is induced by stresses that damage the genome or interfere with its replication. The fraction of cells exhibiting nucleoid compaction was proportional to the dose of the DNA damaging agent, and results obtained in cells defective for nucleotide excision repair suggest that breakage of DNA strands triggers reorganization of the nucleoid. We observed that compaction depends on the Mre11-Rad50 complex, suggesting a link to DNA double-strand break repair. However, compaction was observed in a *radA* mutant, indicating that the role of Mre11-Rad50 in nucleoid reorganisation is independent of homologous recombination. We therefore propose that nucleoid compaction is part of a DNA damage response that accelerates cell recovery by helping DNA repair proteins to locate their targets, and facilitating the search for intact DNA sequences during homologous recombination.

## Introduction

Cells threatened with stresses that damage the genome or interfere with DNA replication undergo drastic changes in their metabolism. The DNA damage response involves many proteins, which prepare a local environment suitable for DNA repair, manage DNA repair and tolerance pathways, and can arrest the cell cycle (Friedberg *et al*., 2006). In bacteria these DNA transactions occur in the cytoplasm, in which the genome is embedded. The genome is folded and compacted into a dynamic structure named the nucleoid (Luijsterburg *et al*., 2008), which is maintained by DNA supercoiling, molecular crowding, DNA-binding proteins, and transcription (Cabrera and Jin, 2003; Luijsterburg *et al*., 2008; Dillon and Dorman, 2010). The main force that expands the nucleoid is the coupling of transcription, translation and translocation of membrane proteins, called transertion (Norris, 1995; Woldringh *et al*., 1995).

The degree of nucleoid compaction varies as a function of the cellular growth phase and rate (Akerlund *et al*., 1995; Poplawski and Bernander, 1997; Cabrera and Jin, 2003). Moreover, amino-acid starvation that induces the stringent response leads to nucleoid expansion (Cabrera and Jin, 2003; Ferullo and Lovett, 2008), and changes in the structure of the bacterial nucleoid are also observed after DNA damage; the nucleoids of *Bacillus subtilis* and *Escherichia coli* both become significantly more compact after UV irradiation (Smith *et al*., 2002; Odsbu *et al*., 2009). Electron microscopy of *E. coli* following UV irradiation or treatment with the DNA gyrase inhibitor nalidixic acid show reorganization of the DNA into a lateral fibre in a small area of the cytoplasm. The formation of such a structure requires the RecA protein (Levin-Zaidman *et al*., 2000). In *E. coli*, RecA is involved in several aspects of the DNA damage response, such as the induction of the SOS system and homologous recombination. However, it has been proposed that the reorganization of the DNA after DNA damage in *E. coli* does not require the induction of the SOS system (Levin-Zaidman *et al*., 2000). Similarly in *B. subtilis*, RecA is required for compaction of the nucleoid after UV (Smith *et al*., 2002). These examples suggest that nucleoid compaction might be part of a cellular response to environmental or cytotoxic stress.

Like bacteria, archaea do not possess a nucleus and their genome is embedded in the cytoplasm. Analyses of archaeal genomes have revealed a chimera of bacterial and eukaryotic features, the latter being prominent in DNA metabolism and information-processing pathways (Allers and Mevarech, 2005). For example, the archaeal RNA polymerase is structurally similar to eukaryotic RNA polymerase II, but archaeal transcription regulators are more related to bacterial factors (Geiduschek and Ouhammouch, 2005; Facciotti *et al*., 2007; Hirata *et al*., 2008; Korkhin *et al*., 2009). Furthermore, transcription and translation are coupled in archaea, as in bacteria (French *et al*., 2007).

*Haloferax volcanii* is a halophilic archaeon, growing optimally at salt concentrations of 1.7–2.5 M. The genome of *H. volcanii* contains one main chromosome and three mega-plasmids (Hartman *et al*., 2010). Furthermore, *H. volcanii* is polyploid, containing 10–20 copies of its genome depending on the growth phase (Breuert *et al*., 2006). Little is known on how the genome of *H. volcanii* is maintained but we have proposed that the high genome copy number influences the strategy of DNA repair (Delmas *et al*., 2009). We have shown previously that homologous recombination catalysed by the RadA recombinase is required for DNA repair, but that its usage is delayed by Mre11-Rad50 in order to allow other pathways (possibly DNA microhomology-mediated end joining) to act first (Delmas *et al*., 2009). The Mre11-Rad50 complex is present in all domains of life (Aravind *et al*., 1999) and is one of the first proteins to localize at DNA double-strand breaks, where it is proposed to tether DNA ends and assemble a scaffold for DNA processing and repair (de Jager *et al*., 2001; Lisby *et al*., 2004; Moreno-Herrero *et al*., 2005; Williams *et al*., 2008).

In this study we monitored the structure of the nucleoid of *H. volcanii* following cytotoxic stresses. We show that the nucleoid of *H. volcanii* is diffuse throughout the cytoplasm in unperturbed cells growing in exponential phase, and appears compacted into a smaller area following stresses that damage the genome and/or interfere with its replication. This compaction does not depend on homologous recombination but does involve the Mre11-Rad50 complex. We propose that nucleoid reorganization is part of the DNA damage response, facilitating the search for intact DNA sequences during homologous recombination and the localization of DNA repair proteins to sites of damage, thereby accelerating cellular recovery.

## Results

### DNA damage and replication stress trigger nucleoid compaction

**Fig. 1:**
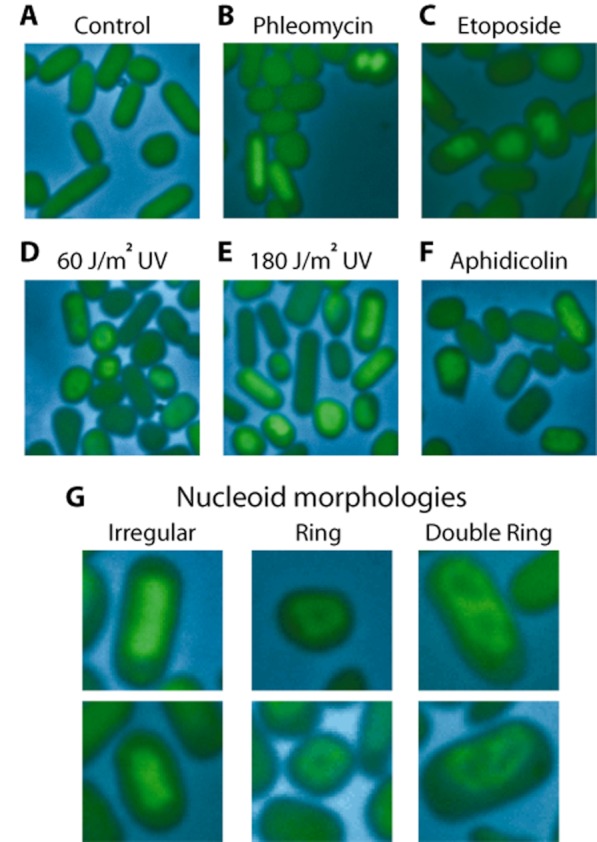
DNA damage and replication arrest induce nucleoid compaction. Fluorescence microscopy images of WT cells (H115) (phase contrast in blue, DNA in green) in (A) exponential phase, (B) and (C) after 1 h in presence of phleomycin or etoposide respectively, (D) and (E) 1 h after irradiation with doses of UV of 60 or 180 J m^−2^ respectively and (F) after 1 h in presence of aphidicolin. G. Diverse morphologies of compacted nucleoids.

We examined the nucleoid structure in *H. volcanii* cells by fluorescence microscopy after staining with the nucleic acid stain acridine orange (AO). In cells growing in exponential phase in complete media (generation time of ∼ 2.5 h), nucleoid staining was observed homogenously throughout the cytoplasm (Fig. 1A). To examine the effect of DNA damaging agents, cells were treated for 1 h with phleomycin, which creates single- and double-stranded breaks in the DNA molecule, or etoposide, an inhibitor of topoisomerase II that leads to double-strand breaks. As may be seen in Fig. 1B and C, we observed that in many cells the nucleoid appeared compacted, with increased fluorescence intensity in a smaller volume of the cell. Compaction of the nucleoid was also seen after UV irradiation at doses of 60 or 180 J m^−2^ (Fig. 1D and E) and treatment with the reversible DNA replication inhibitor aphidicolin (Fig. 1F). The compacted nucleoids usually took on a dense irregular shape, but ‘ring’ and ‘double-ring’ nucleoid morphologies were also observed, mainly after aphidicolin treatment or 60 J m^−2^ UV (Fig. 1G). We use the term ‘compacted’ to describe this general phenotype, but note that the physical processes of nucleoid reorganization and/or increased dye-accessibility could cause this effect.

**Fig. 2:**
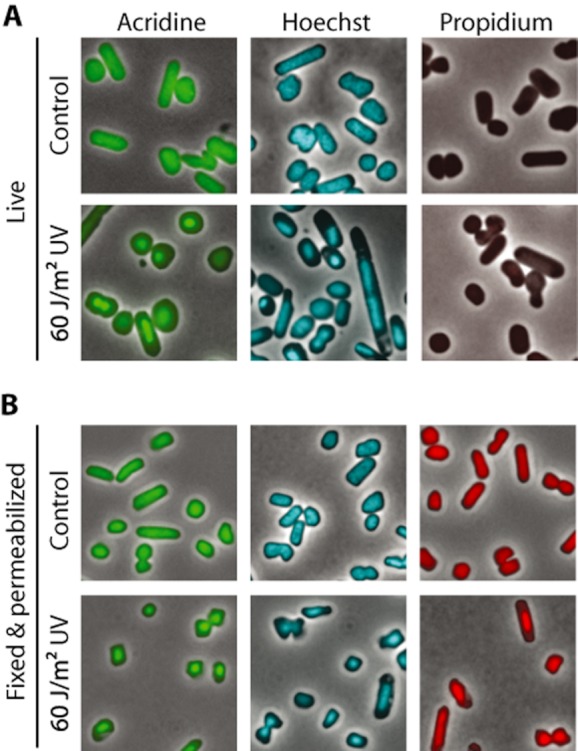
*H. volcanii* responds to UV irradiation by nucleoid compaction. Mid-log phase WT cells (H26) were (A) stained directly with the nucleic acid stains acridine orange, Hoechst 33342, or propidium iodide, or (B) formaldehyde fixed and then ethanol permeabilized before staining. Results for both control and UV-treated (60 J m^−2^) cells are shown. The compaction effect is independent of the choice of stain and cell permeability.

To test whether nucleoid compaction was an effect of the particular DNA dye chosen (AO) or a change in cell permeability, we also used the membrane-permeable dye Hoechst 33342 or the membrane-impermeable dye propidium iodide. Similar to the results obtained with AO, compaction of the nucleoid was observed after UV irradiation in unfixed cells stained with Hoechst 33342, whereas the nucleoid was spread throughout the cytoplasm in non-irradiated exponential-phase cells (Fig. 2A). No staining was observed in untreated cells (irradiated or non-irradiated) using propidium iodide (Fig. 2A). However, in cells that had been fixed with formaldehyde and then permeabilized with ethanol, staining of nucleoid was observed with all three dyes (Fig. 2B). In each case the nucleoid was spread throughout the cytoplasm in untreated cells, whereas the nucleoid became compacted after UV irradiation (Fig. 2B). These results indicate that the nucleoid compaction we observe after UV irradiation is independent of the choice of stain and cell permeability. We conclude that treatment of *H. volcanii* with these genotoxic agents, which lead to DNA damage and/or arrest of DNA replication, results in a genuine change in nucleoid structure.

These genotoxic agents ultimately lead to cell death in a dose-dependent manner, as measured by the failure to form a colony. For example, after UV doses of 60 or 180 J m^−2^, 60% and 0.1% of cells are able to form a colony respectively (Delmas *et al*., 2009). Therefore, the population that we observe with a compacted nucleoid would be a mixture of survivor and moribund individuals. We assume that the latter are not yet dead at time of microscopy, because they respond by nucleoid compaction and remain impermeable to propidium iodide (Fig. 2A). Furthermore, we found that treatment with the DNA replication inhibitor aphidicolin induces pronounced nucleoid compaction (Fig. 1F), when used at a dose that blocks DNA synthesis within 5 min (as determined by incorporation of ^3^H thymidine). However, aphidicolin treatment leads to no detectable cell death (after 3 h, data not shown) suggesting that nucleoid compaction is not a hallmark of moribund cells.

### Effect of DNA damaging agent dose on nucleoid compaction

**Fig. 3:**
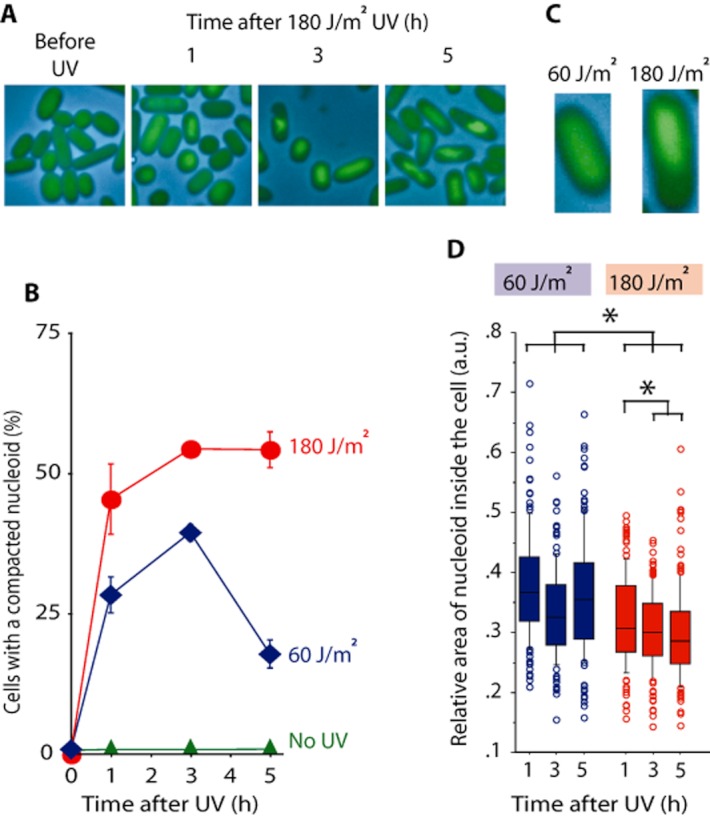
Nucleoid compaction depends on dose of DNA damage. A. Fluorescence microscopy images (phase contrast in blue, DNA in green) of WT (H115) cells before and after irradiation with 180 J m^−2^ UV. B. Percentage of cells having a compacted nucleoid during the recovery after irradiation with 0, 60 or 180 J m^−2^ UV as determined from microscopy images. Each point represents the mean (± SEM) values of at least three independent experiments. At least 300 cells were counted per time point per experiment. C. Fluorescence microscopy images of WT cells (H115) (phase contrast in blue, DNA in green) after irradiation with doses of 60 or 180 J m^−2^ UV showing different degrees of nucleoid compaction. D. Relative area occupied by the compacted nucleoid inside cells during the recovery after irradiation with doses of 60 or 180 J m^−2^ UV. At least 121 individual cells were analysed; the asterisk corresponds to *P* < 0.05 by unpaired Student's *t*-test.

We analysed the effect of different doses of DNA damage on nucleoid compaction by irradiating cells with 0, 60 or 180 J m^−2^ UV. When growing unperturbed in exponential phase, ∼ 0.3% of cells contain a compacted nucleoid (Fig. 3A and B). After both doses of UV, the percentage of cells in the population with a compacted nucleoid increased for 3 h after irradiation (to 38% and 55% after UV doses of 60 or 180 J m^−2^ respectively) (Fig. 3B). At a dose of 60 J m^−2^, the fraction of cells with a compacted nucleoid declined to 18% after 5 h post-irradiation, while at a dose of 180 J m^−2^, this fraction of cells remained stable (Fig. 3B). Significantly, the percentage of cells with a compacted nucleoid was higher and persisted for longer after 180 J m^−2^ than 60 J m^−2^ UV. It is uncertain why not all the cells show nucleoid compaction after genotoxic stress that affects more than 99.9% of the population. One possibility is that only cells in a specific phase of the cell cycle are able to compact their nucleoid. To determine the degree of compaction, we estimated the area occupied by the compacted nucleoid within the cytoplasm. The nucleoid appeared more compact after 180 J m^−2^ than 60 J m^−2^ UV (Fig. 3C and D); after a dose of 180 J m^−2^ UV, the area occupied by the nucleoid decreased over time (Fig. 3D). These results indicate that the cellular response leading to a compacted nucleoid, and the degree of compaction, depend on the dose of DNA damage.

### Nucleoid compaction determined by flow cytometry

**Fig. 4:**
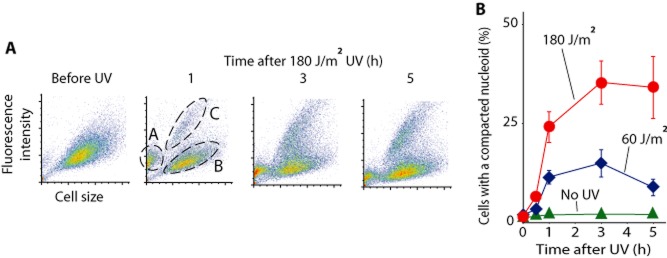
Nucleoid compaction detected by flow cytometry. A. Flow cytometry analysis of cell size versus acridine orange fluorescence intensity after irradiation of WT *H. volcanii* (H115) with a dose of 180 J m^−2^ UV. At 1 h after UV irradiation, the three populations (A, B and C) described in the results are highlighted. B. Percentage of population C among populations C and B during the recovery, after irradiation with doses of UV of 0, 60 or 180 J m^−2^. Each point represents the mean (± SEM) values of at least four independent experiments.

Since the fluorescence of cells with a compacted nucleoid was more intense, we hypothesized that these cells could be detected using flow cytometry. In exponential growth phase, a plot of fluorescence intensity against scatter (cell size) shows a single population, with a positive linear relationship between both parameters (Fig. 4A). After UV irradiation, three populations were observed (Fig. 4A). The population labelled ‘A’ is of small size and might represent virus-like membrane vesicles as reported for several archaeal species (Roine *et al*., 2010; Soler *et al*., 2011), and/or consist of debris and dead cells. The two other populations (labelled ‘B’ and ‘C’) exhibit the same range of cell size as the unirradiated sample, and are differentiated by their fluorescence intensity (Fig. 4A). The population ‘B’ shows a decrease in fluorescence intensity and might correspond to the darker cells observed on the microscope images (Fig. 1); this decrease in fluorescence might be due to DNA degradation, as suggested by the chromosomal fragmentation (by double-stranded DNA breakage) that we observe within 30 min of UV irradiation (Fig. S1). The population ‘C’ is of higher fluorescence intensity and most probably represents cells with a compacted nucleoid, since the percentage of cells with a compacted nucleoid seen under the microscope and the percentage of the population ‘C’ as determined by flow cytometry show a similar trend (compare Figs 3B and 4B). The discrepancy in numerical values between these two methods is most likely due to the difference in fluorescence intensity thresholds that could be discriminated microscopically and by flow cytometry. These results suggest that flow cytometry can be used along with microscopy, as a high-throughput approach to measure the percentage of cells with a compacted nucleoid.

### Mre11-Rad50 dependent pathway of nucleoid compaction

**Fig. 5:**
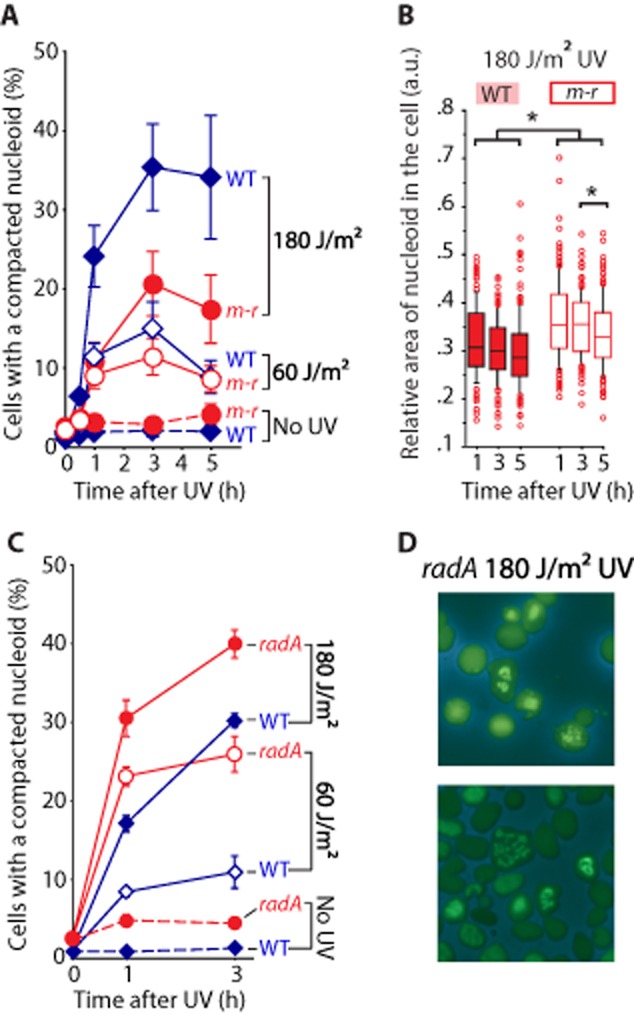
Nucleoid compaction depends on Mre11-Rad50 but not homologous recombination. A. Percentage of cells with a compacted nucleoid determined by flow cytometry in WT (H115) and *mre11 rad50* (*m-r*) mutant (H204) after irradiation with 0, 60 and 180 J m^−2^ UV. Each point represents the mean (± SEM) values from at least three independent experiments. B. Relative area occupied by the compacted nucleoid inside WT (H115) and *mre11 rad50* (*m-r*) (H204) cells after irradiation with 60 or 180 J m^−2^ UV. At least 121 individual cells had been analysed, the asterisk corresponds to *P* < 0.05 by unpaired Student's *t*-test. C. Represents the percentage of cells with a compacted nucleoid determined by flow cytometry in WT (H115) or Δ*radA* mutant (H607) after irradiation with doses of UV of 0, 60 or 180 J m^−2^. D. Fluorescence microscopy images (phase contrast in blue, DNA in green) of *ΔradA* cells (H607) 3 h after irradiation with 180 J m^−2^ UV.

In *H. volcanii* the Mre11-Rad50 complex is required for rapid cell recovery after UV irradiation (Delmas *et al*., 2009). We examined the role of Mre11-Rad50 in nucleoid compaction after UV irradiation using an *mre11 rad50* deletion mutant. Flow cytometry and microscope image analysis showed that UV-induced nucleoid compaction takes place in an *mre11 rad50* mutant (Fig. 5A). However, at doses of both 60 and 180 J m^−2^ UV, fewer cells with a compacted nucleoid were present in the *mre11 rad50* mutant than the WT (wild type), indicating that nucleoid compaction is partially dependent on Mre11-Rad50 (Fig. 5A). Complementation of *mre11 rad50* deletion by expressing this operon on a replicative plasmid led to an increase in nucleoid compaction (compared with the control strain carrying an empty vector), confirming that Mre11-Rad50 is involved in reorganization of the nucleoid after DNA damage (Fig. S2A). We estimated the degree of compaction of the nucleoid by measuring the area it occupied inside the cytoplasm. Following 60 J m^−2^ UV, the area occupied by the compacted nucleoid showed no difference between the WT and mutant (data not shown), while at a UV dose of 180 J m^−2^ the area occupied by the nucleoid was smaller in WT than the *mre11 rad50* mutant (Fig. 5B). This result suggests that Mre11-Rad50 increases the degree of compaction of DNA inside the nucleoid.

### Homologous recombination is not required for nucleoid compaction

We examined the role of homologous recombination in nucleoid compaction, using a strain deleted for *radA*. In archaea, RadA is the orthologue of the RecA/Rad51 recombinase. *H. volcanii radA* mutants are slow growing, show increased sensitivity to DNA damaging agents, and are deficient in homologous recombination (Woods and Dyall-Smith, 1997). When grown unperturbed, *radA* mutants show a significantly higher proportion of cells (∼ 3%) with a compacted nucleoid compared with the WT (Fig. 5C). Flow cytometry and microscope image analyses showed that irradiation with 60 or 180 J m^−2^ UV led to nucleoid compaction in the mutant (Fig. 5C and D); at all time points tested, more cells with a compacted nucleoid are observed in the *radA* mutant than the WT (Fig. 5C). Analysis of microscope images revealed that the structure of the compacted nucleoid is also different in the *radA* mutant; cells can be observed with fragments of a compacted nucleoid that are dispersed in the cytoplasm (Fig. 5D). This phenotype was reversed by complementation of *radA*, by expressing the wild-type *radA* gene from a replicative plasmid (Fig. S2B). These results suggest that homologous recombination is not required to compact the nucleoid after DNA damage; however, it is required to maintain a discrete compacted morphology.

### Inhibition of nucleoid compaction by actinomycin D, and stimulation by anisomycin

**Fig. 6:**
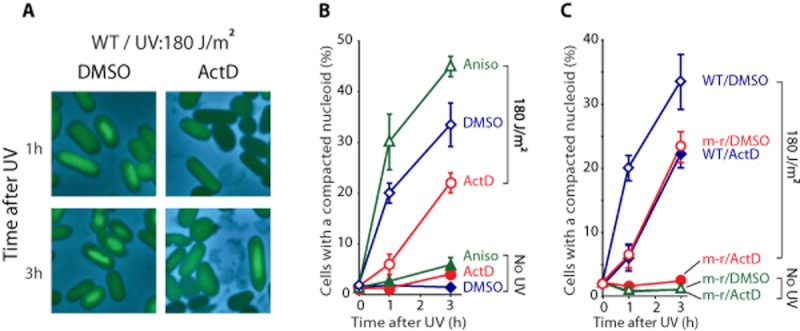
Actinomycin D inhibition of nucleoid compaction. A. Fluorescence microscopy image (phase contrast in blue, DNA in green) of WT cells (H115) after irradiation with 180 J m^−2^ UV in the presence of DMSO or actinomycin D. B. Percentage of WT cells (H115) with a compacted nucleoid determined by flow cytometry after irradiation with 0 or 180 J m^−2^ UV in the presence of DMSO, anisomycin or actinomycin D. Each point represents the mean (± SEM) values from three independent experiments. C. Percentage of WT (H115) and *mre11 rad50* (*m-r*) mutant (H204) cell with a compacted nucleoid determined by flow cytometry after irradiation with 0 and 180 J m^−2^ UV in the presence of DMSO or actinomycin D. Each point represents the mean (± SEM) values from three independent experiments.

Actinomycin D (ActD) is an antibiotic that is known to block DNA replication and transcription (Jager *et al*., 2002). As determined by both microscopy and flow cytometry (Fig. 6A and B), ActD treatment reduced the fraction of WT cells with a compacted nucleoid seen 1 h after UV irradiation [5% of the population, compared with 20% in the dimethylsulphoxide (DMSO)-treated control]. Three hours after UV irradiation, the fraction of ActD-treated cells with a compacted nucleoid increased to 22% (Fig. 6A and B). These results indicate that ActD prevents compaction of the nucleoid within the first hour after UV irradiation. This delay could be due to the ActD-mediated absence of protein(s) that are induced by DNA damage and stimulate nucleoid compaction. To test this possibility, we followed nucleoid compaction in presence of the translation inhibitor anisomycin (Mankin *et al*., 1992). In unirradiated cells we observed a slight increase in the percentage of cells with a compacted nucleoid (∼ 5% of the total population), but only after 5 h after treatment with anisomycin. This suggests that preventing translation, and by inference transertion, does not cause nucleoid compaction in *H. volcanii* (Fig. 6B). In cells that were UV-irradiated and left to recover in the presence of anisomycin, an increased fraction of UV-irradiated cells exhibited a compacted nucleoid: 1 h after irradiation, 30% of cells treated with anisomycin showed a compacted nucleoid, while in the control cells this fraction was 22% (Fig. 6B). These results show that compaction of the nucleoid after DNA damage does not require *de novo* protein synthesis. We then tested the effect of ActD in an *mre11 rad50* mutant. When the *mre11 rad50* mutant was challenged with ActD after UV irradiation, the DNA damage-induced nucleoid compaction was completely abrogated (Fig. 6C). These results suggest that there are two pathways to reorganize the nucleoid after DNA damage, one that is dependent on Mre11-Rad50 and another that is sensitive to ActD.

### Nucleotide excision repair influences nucleoid compaction

**Fig. 7:**
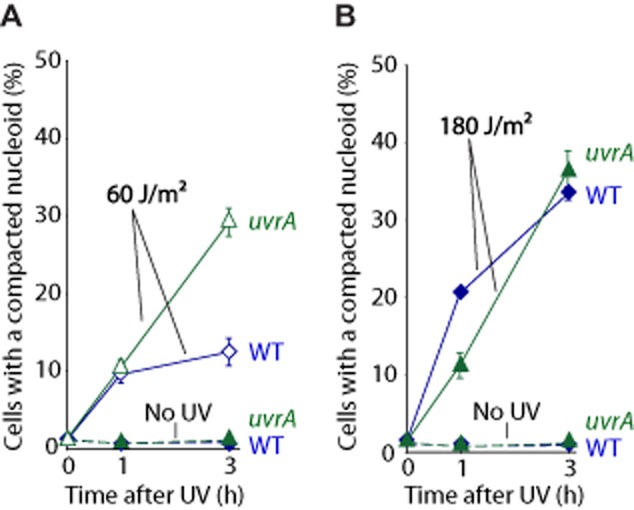
Role of nucleotide excision repair in nucleoid compaction. (A) and (B) represent the percentage of cells with a compacted nucleoid determined by flow cytometry in WT (H26) and Δ*uvrA* mutant (H509) after irradiation with 0, 60 or 180 J m^−2^ UV. Each point represents the mean (± SEM) values from four independent experiments.

The primary mechanism to repair UV-induced lesions is the nucleotide excision repair pathway (NER) (Friedberg *et al*., 2006). *H. volcanii* uses NER that is homologous to the bacterial UvrABC pathway (Lestini *et al*., 2010). We tested the role of NER in nucleoid compaction by irradiating a strain deleted for *uvrA*. The fraction of cells with a compacted nucleoid was similar in both WT and mutant cells when growing unperturbed in exponential phase (Fig. 7A and B). After 60 J m^−2^ or 180 J m^−2^ UV, the percentage of cells with a compacted nucleoid increased in the *uvrA* mutant (Fig. 7A and B). Flow cytometry and microscope image analyses indicated that 3 h after irradiation with 60 J m^−2^ UV, more *uvrA* mutant cells exhibited a compacted nucleoid than the WT (29% compared with 12%) (Fig. 7A). Three hours after 180 J m^−2^ UV there was no difference between both strains, but 1 h after irradiation fewer *uvrA* mutant cells showed nucleoid compaction than the WT (11% compared with 20%) (Fig. 7B). These results suggest that NER influences the signal that triggers nucleoid compaction.

## Discussion

In this study we show that in the archaeon *H. volcanii*, stress that damages DNA and/or blocks its replication induces reorganization of the nucleoid; this process does not require *de novo* protein synthesis. Our results indicate that the Mre11-Rad50 complex is involved in nucleoid compaction after genomic stress. We show that fewer cells exhibit a compacted nucleoid in the *mre11 rad50* mutant compared with the WT (Fig. 5A), and that the area occupied by the compacted nucleoid inside the cytoplasm is smaller in the WT than the *mre11 rad50* strains (Fig. 5B). Mre11-Rad50 might recruit proteins that target the chromatin and bring about compaction of the nucleoid, and might also play a direct role in maintaining the dense structure of the compacted nucleoid by tethering broken DNA fragments, thereby keeping double-strand breaks in close proximity (de Jager *et al*., 2001; Costanzo *et al*., 2004; Lobachev *et al*., 2004; Moreno-Herrero *et al*., 2005). The role of Mre11-Rad50 in nucleoid compaction in *H. volcanii* is independent of homologous recombination, since nucleoid compaction was observed in a *radA* mutant (Fig. 5C). This result is in contrast to what has been observed in bacteria, where nucleoid compaction is dependent on RecA and has been proposed to rely on its recombinase function (Levin-Zaidman *et al*., 2000; Smith *et al*., 2002). However, an active role of homologous recombination in maintaining the nucleoid in a compacted state is suggested by the phenotype of nucleoid fragmentation observed in the *radA* deletion strain (Fig. 5D).

A second pathway of nucleoid compaction that is sensitive to ActD is also active in *H. volcanii* (Fig. 6A and B). ActD stalls both transcription and DNA replication by intercalating into the DNA template. It is unlikely that blocking DNA replication in this manner prevents nucleoid compaction; we show that inhibiting DNA replication by using aphidicolin actually induces nucleoid compaction (Fig. 1F). Transcription is one candidate for the ActD-sensitive pathway. It has been proposed that active transcription may play multiple roles in the cellular organization and dynamics of chromosomes in both the cytoplasm of prokaryotes and nucleus of eukaryotes (Cook, 1999; Cabrera and Jin, 2003; Kruse *et al*., 2006; Soutoglou and Misteli, 2007). For example, increased transcription of loci around the chromosome has been proposed to bring about their clustering in transcription factories and to contribute to nucleoid compaction. Nucleoid-associated proteins have also the potential to create bridges between distant loci around the chromosome hence compacting the DNA, and ActD could prevent their binding (Luijsterburg *et al*., 2008). Indeed, DNA binding by the nucleoid-associated protein GroEL1 of *Mycobacterium tuberculosis* is inhibited by ActD (Basu *et al*., 2009).

The mode of action of the different genotoxic agents that trigger nucleoid compaction are wide-ranging, but results obtained with NER-deficient mutant suggest that single and double-stranded DNA breaks trigger nucleoid compaction. At a dose of 60 J m^−2^ UV, more cells with a compacted nucleoid were observed in the *uvrA* mutant compared with the WT, while at a higher dose of UV, the mutant had fewer cells with a compacted nucleoid in the first hour after irradiation (Fig. 7A and B). At lower UV doses, UV-induced lesions impact directly on DNA replication; unrepaired DNA lesions in the *uvrA* mutant increase the probability that the DNA replication machinery encounter a lesion and stalls, resulting in a single-strand gap (Rupp and Howard-Flanders, 1968; Sassanfar and Roberts, 1990; Heller and Marians, 2006). At higher UV doses, it has been shown that chromosome fragmentation is brought about by the activity of NER, since the excision of lesions that are in close proximity leads to a double-stranded DNA break (Bonura and Smith, 1975; Thoms and Wackernagel, 1998) (see also Fig. S1). The hypothesis that unrepaired DNA breaks are the signal for the induction of nucleoid compaction in *H. volcanii* is reinforced by our observation that a *radA* strain deficient in homologous recombination (the primary pathway for double-strand break repair) shows more nucleoid compaction than the WT, even when grown unperturbed (Fig. 5C).

The results presented in this study suggest that in the archaeon *H. volcanii*, the Mre11-Rad50 complex acts as part of a cell-wide DNA damage response, as seen in eukaryotes (Williams *et al*., 2007; Delmas *et al*., 2009). The reorganization of the nucleoid of *H. volcanii* after DNA breakage might facilitate the search for intact DNA sequences during the process of homologous recombination, similar to what has been recently proposed in yeast (Dion *et al*., 2012; Mine-Hattab and Rothstein, 2012). Nucleoid compaction might also accelerate the localization of repair proteins to sites of DNA damage through 3D diffusion (van den Broek *et al*., 2008). The increased fluorescence intensity of the compacted nucleoid in *H. volcanii* indicates an increased accessibility to DNA dyes, suggesting that the chromatin might also be more accessible to proteins involved in cellular recovery. The requirement for the Mre11-Rad50 complex in nucleoid reorganization, co-ordination of DNA repair pathways and rapid cell recovery after DNA damage suggests that all these process are linked in *H. volcanii* (Delmas *et al*., 2009).

## Experimental procedures

### Strains

*Haloferax volcanii* strains and plasmids used in this study are described in Table 1.

**Table 1:** Strains and plasmids used

Strain	Genotype^a^	Plasmid^b^	Reference
H26	Δ*pyrE2*		Allers *et al*. (2004)
H115	Δ*pyrE2 bgaHa-Kp*		Delmas *et al*. (2009)
H509	Δ*pyrE2 ΔuvrA*		Lestini *et al*. (2010)
H204	Δ*pyrE2 bgaHa-Kp* Δ*mre11* Δ*rad50*		Delmas *et al*. (2009)
H607	Δ*pyrE2 bgaHa-Kp* Δ*trpA* Δ*radA*::*trpA*^+^		Delmas *et al*. (2009)
H380	*ΔpyrE2 ΔradA* p{*radA^+^ pyrE2^+^ hdrB^+^*}	pTA411	This study^c^
H385	ΔpyrE2 *ΔradA* p{*pyrE2+ hdrB+*}	pTA409	This study^c^
H732	Δ*pyrE2 bgaHa-Kp* Δ*mre11* Δ*rad50* p{*pyrE2^+^*}	pTA354	Delmas *et al*. (2009)
H739	Δ*pyrE2 bgaHa-Kp* Δ*mre11* Δ*rad50* p{*mre11-rad50^+^ pyrE2^+^*}	pTA795	Delmas *et al*. (2009)

a. Genes within p{…} are plasmid-encoded.

b. pTA354 is described in Guy *et al*. (2006), pTA409, pTA411 and pTA795 are described in Delmas *et al*. (2009).

c. All *ΔradA* strains are derived from H112 (Norais *et al*., 2007).

### Media and antibiotics

Strains were grown at 45°C. Complete (Hv-YPC) agar or broth (Allers *et al*., 2004) was used for growing strains H26, H115, H509, H204 and H607. Hv-Ca agar or broth (Guy *et al*., 2006) was used to grow strains H380, H385, H372 and H379. Where appropriate, dyes/antibiotics were added to cell cultures and used at the following concentrations: AO (10 μg ml^−1^, Sigma-Aldrich), Hoechst 33342 (1 μg ml^−1^), propidium iodide (1 μg ml^−1^), phleomycin (2 mg ml^−1^, Apollo Scientific), and from Alexis Biochemicals, aphidicolin (200 μg ml^−1^), etoposide (50 μg ml^−1^), anisomycin (10 μg ml^−1^) and ActD (50 μg ml^−1^).

### Post-UV recovery assay in broth

For microscopy and flow cytometry, cells were irradiated and left to recover as follows: *H. volcanii* cultures were grown to ∼ 10^8^ cells per ml, centrifuged, and resuspended in an equal volume of 18% SW (salt water). Aliquots were either exposed in the dark to 60 or 180 J m^−2^ UV with shaking, or left unirradiated. Cells were centrifuged again, resuspended in an equal volume of Hv-YPC broth (containing, if required, antibiotic or solvent control) and grown in the dark at 45°C with agitation.

### Microscopy

Fifty-microlitre samples were taken at the time indicated, centrifuged, resuspended in 18% SW and stained with AO for 5 min. Cells were mounted on glass slides covered with a thin layer of 1% agarose prepared in 18% SW. Cells were visualized using a BX-52 Olympus microscope equipped with a 100× oil immersion objective and a coolSNAP™HQ camera (Photometrics), using the JP4-CFP-YFP filterset 86002v2 (Chroma). Images were taken in phase contrast and in fluorescence, analysed using MetaMorph 6.2 (Universal Imaging), and processed using MetaMorph and Adobe Photoshop. For each time point at least three different fields on the agarose slide had been taken for image analysis. ImageJ (NIH) was used to estimate the area occupied by the compacted nucleoid inside the cell, by approximating a rectangle for both the cell and nucleoid size. For Fig. 2, live cells in the mid-log growth phase were centrifuged, and then resuspended in 18% SW, containing either AO, Hoechst 33342, or Propidium iodide. One microlitre of the cell suspension was then placed onto an agarose pad (1% agarose, 18% SW, and the appropriate stain, above) on a microscope slide, and then covered with a coverslip. Images were acquired on a Zeiss Axioplan 2 epifluorescence microscope, with a phase-contrast 100×/1.4 lens and FITC, DAPI or Rhodamine filter sets for fluorescence detection. Phase contrast (greyscale) and fluorescence (pseudo-colour) images were acquired for each field and these were overlaid, with linear contrast adjustment, using Adobe Photoshop.

### Cell fixation

For fixation, cells were first centrifuged, and then resuspended in freshly prepared 4% formaldehyde, 18% SW (except 10 mM HEPES, pH 7.5 replaced the Tris). Cells were incubated for 2 h at room temperature, and then were centrifuged and resuspended in 18% SW. To permeabilize the cells, 70% ethanol was added to the cells at room temperature to give a final ethanol concentration of 60%, and the cells were mixed immediately. The cells were then centrifuged and washed with 18% SW, before final resuspension in 18% SW plus the appropriate stain, and were prepared for microscopy as described above.

### Flow cytometry

Samples were taken at the times indicated and stained with AO as for microscopy. Flow cytometry was performed using an Apogee A40 equipped with a 50 mW 488 nm solid state laser (Coherent) and a 510–580 nm bandpass filter as described previously (Breuert *et al*., 2006). Latex beads of a uniform size and fluorescence were used for calibration and adjustment of the flow cytometer. Doublet signals were removed by gating on peak/area plots for LS1 and FL1. For each time point 50 000 cells were analysed. Calculations were carried out using FlowJo (TreeStar).
