# Supplementary material for: DNA damage induces nucleoid compaction via the Mre11-Rad50 complex in the archaeon Haloferax volcanii
Source: Mol Microbiol. 2012 Nov 30;87(1):168–79. doi: 10.1111/mmi.12091 (PMC3565448; doi:10.1111/mmi.12091)
Supplement: Supplementary file 1 [file mmi0087-0168-SD1.pdf]

Supplementary Figure 1: UV irradiation leads to genome fragmentation

Samples of WT cell (H115) were taken immediately before (unirradiated, Unir.) and at 0, 10 and 30 minutes after irradiation with 180 J/m^2 UV. Genomic DNA in agarose plugs was digested with *Pme*I and subject to pulsed-field gel electrophoresis as described in (Delmas et al., 2009).

Supplementary Figure 2: Complementation of *mre11 rad50* and *radA* deletion.

(A) Percentage of cells with a compacted nucleoid determined by flow cytometry in H739 (H204 p{*mre11rad50+*}) and H732 (H204 *empty vector*) after irradiation with 0, 60 and 180 J/m^2 UV. Each point represents the mean (± SEM) values from 3 independent experiments. (B) Fluorescence microscopy images (phase contrast in blue, DNA in green) of H380 (p{*radA+*}) and H385 (*empty vector*) cells 3 hours after irradiation with 180 J/m^2 UV.

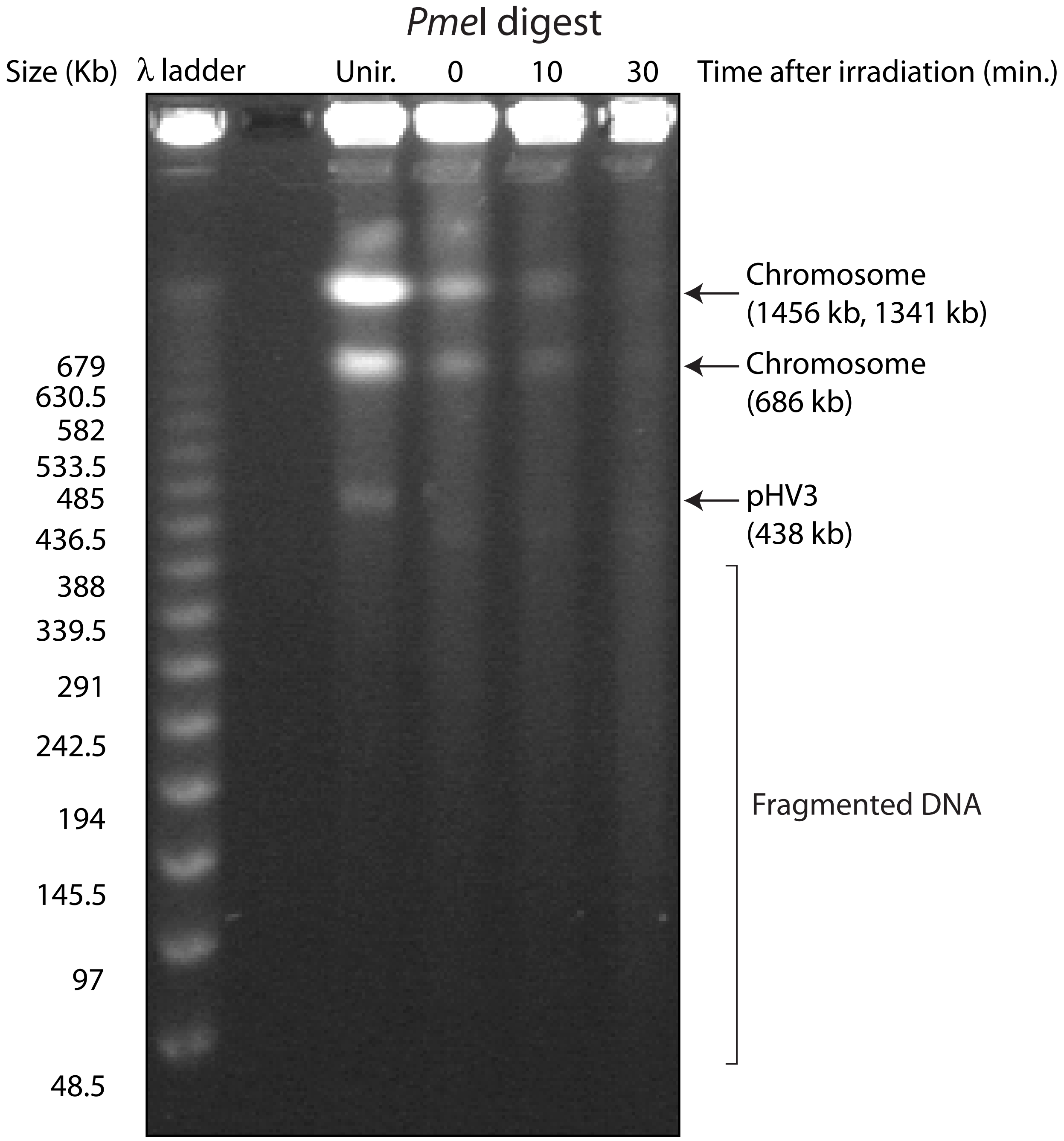

Supplementary Figure 1

A

Complementation of *mre11 rad50*

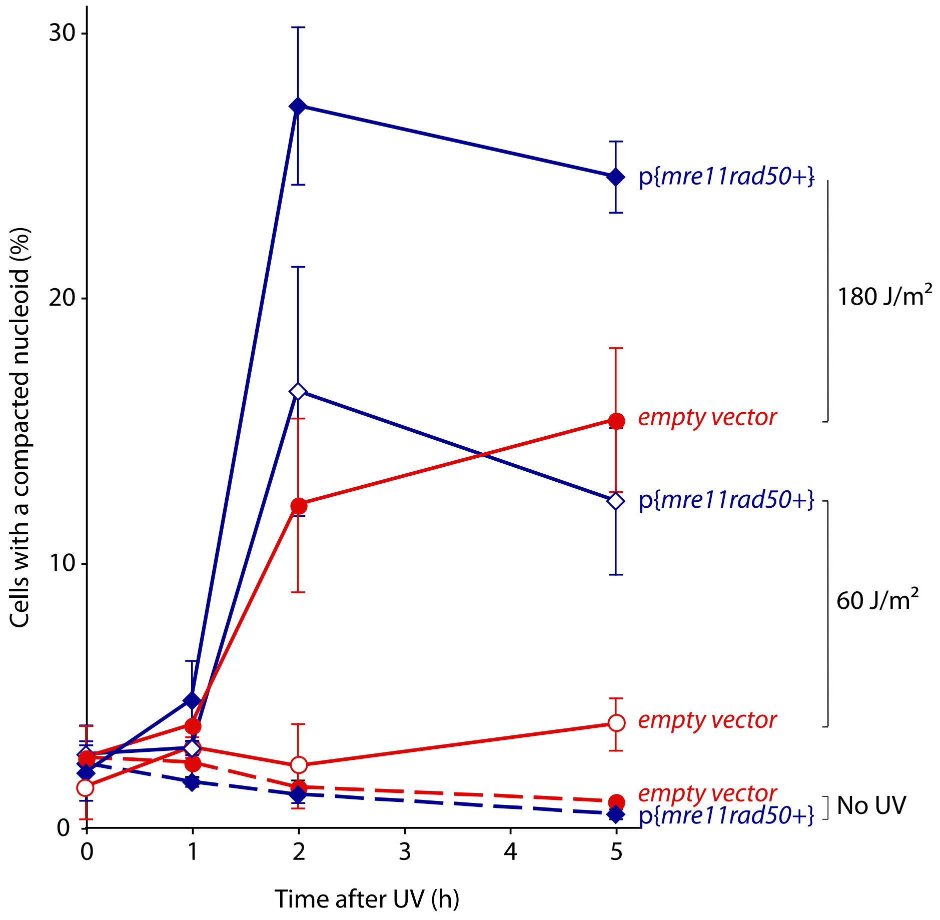

B

Complementation of *radA*

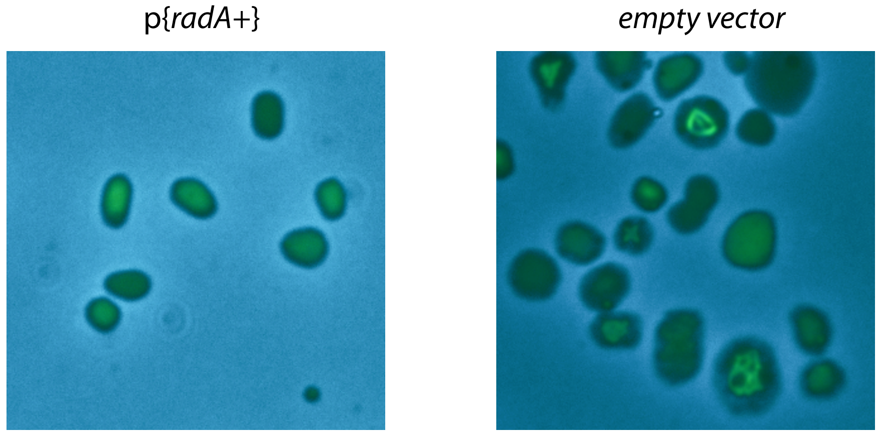

Supplementary Figure 2
